# Holo-Omics: Integrated Host-Microbiota Multi-omics for Basic and Applied Biological Research

**DOI:** 10.1016/j.isci.2020.101414

**Published:** 2020-07-25

**Authors:** Lasse Nyholm, Adam Koziol, Sofia Marcos, Amanda Bolt Botnen, Ostaizka Aizpurua, Shyam Gopalakrishnan, Morten T. Limborg, M.Thomas P. Gilbert, Antton Alberdi

**Affiliations:** 1 Center for Evolutionary Hologenomics, GLOBE Institute, University of Copenhagen, Copenhagen 1353, Denmark; 2 Department of Genetics, Physical Anthropology and Animal Physiology, University of the Basque Country (UPV/EHU), Leioa 48940, Spain; 3 Department of Health Technology, Section for Bioinformatics, Technical University of Denmark, Kongens Lyngby 2800, Denmark; 4 Norwegian University of Science and Technology, University Museum, Trondheim 7491, Norway

**Keywords:** Microbiome, Evolutionary Biology

## Abstract

From ontogenesis to homeostasis, the phenotypes of complex organisms are shaped by the bidirectional interactions between the host organisms and their associated microbiota. Current technology can reveal many such interactions by combining multi-omic data from both hosts and microbes. However, exploring the full extent of these interactions requires careful consideration of study design for the efficient generation and optimal integration of data derived from (meta)genomics, (meta)transcriptomics, (meta)proteomics, and (meta)metabolomics. In this perspective, we introduce the holo-omic approach that incorporates multi-omic data from both host and microbiota domains to untangle the interplay between the two. We revisit the recent literature on biomolecular host-microbe interactions and discuss the implementation and current limitations of the holo-omic approach. We anticipate that the application of this approach can contribute to opening new research avenues and discoveries in biomedicine, biotechnology, agricultural and aquacultural sciences, nature conservation, as well as basic ecological and evolutionary research.

## Graphical Abstract

**Figure:**
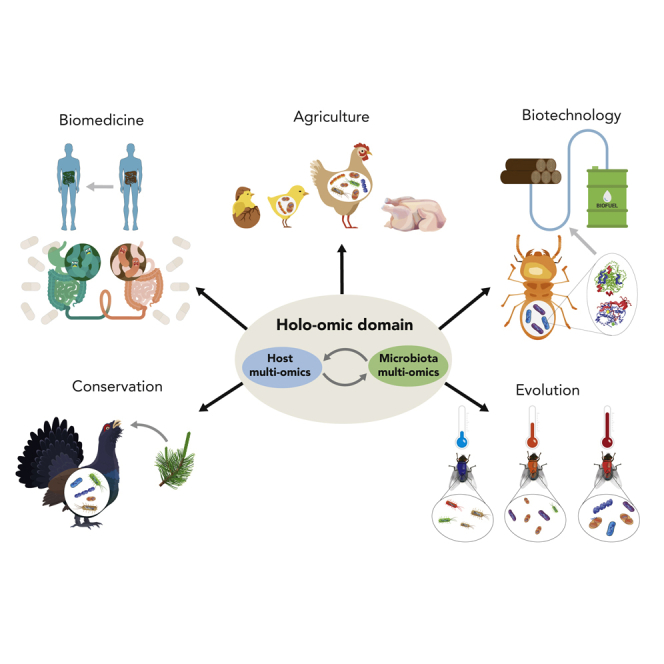

Research conducted over the last decade has fundamentally changed how we perceive the biology and underlying genetic properties of macroorganisms, from looking at individuals as isolated genetic entities to recognizing how they interact with their associated microorganisms in a myriad of biological processes. These microorganisms associated with plants and animals are now acknowledged as relevant—even essential—assets to many basic biological processes, including nutrient acquisition (Falcinelli et al., 2015), immune response (Wu and Wu, 2012), development (Rudman et al., 2019), biomolecule synthesis (Nicholson et al., 2012), and behavior (Liang et al., 2018). This realization has promoted the notion of the **holobiont** (see Box 1 for definitions of this and other terms in bold), a term used to collectively describe the host organism and all its associated microorganisms.

Box 1 **Glossary**

**Amplicon sequencing:**PCR amplification-based targeted sequencing of a specific genetic region.
**Dysbiosis:**Any change to the components of resident commensal microbial communities relative to the community found in healthy individuals.
**Epigenome:**The heritable alteration of DNA or proteins associated with DNA that changes gene expression levels in a cell or tissue without modifying the sequence of DNA.
**Epigenotype**:The pattern of epigenetic modification (alteration of DNA or proteins that changes gene expression) in a cell or tissue.
**Exposome:**Every exposure that an organism is subjected to throughout its lifetime.
**Genome:**The complete set of genetic material of an organism.
**Genome-wide association study**(**GWAS):**An examination of a genome-wide set of genetic variations associated with a trait of interest.
**Holobiont:**A host organism and its associated microorganisms.
**Hologenome:**The combined genetic content of the host and its associated microbiota.
**Holo-omics:**The analysis of multiple omic levels from both host and associated microbiota domains.
**Hologenome theory of evolution:**The theory that posits host, symbionts, and their associated hologenome, acting in consortium, function as a biological entity and as a level of selection in evolution.
**Metagenome-assembled genome (MAG):**Genome assembled from shotgun sequencing data generated from the entire genetic content present in a given environment.
**Metabolome:**The entire pool of metabolites present in an organism.
**Metagenome:**The entire genetic content present in a given environment.
**Metametabolome:**The entire pool of metabolites present in an environmental sample.
**Metaproteome:**The complete set of proteins/peptides present in an environmental sample.
**Metatranscriptome:**The entire pool of mRNA in an environmental sample.
**Metagenome-wide association study (MGWAS):**An examination of a metagenome-wide set of genetic variations associated with a trait of interest.
**Microbiome:**The sum of genetic material in a microbial community.
**Microbiota:**The ecological community of microorganisms.
**Multi-omics:**The analysis of multiple types of omic data (e.g., metagenome and metaproteome).
**Omic:**Term used to describe any level of multi-omics (i.e., (meta)genomics, epigenomics, (meta)transcriptomics, (meta)proteomics, and (meta)metabolomics).
**Proteome:**The entire pool of proteins present in an organism.
**Shotgun DNA sequencing:**The non-targeted sequencing of the entire genetic content of a sample.
**Shotgun proteomics:**The direct analysis of complex protein mixtures to generate global profiles of proteins within a sample.
**Single cell sequencing:**Sequencing of the nucleic acid content within a single cell.
**Spatial metagenomics:**Characterization of the spatial orientation of microbes in their environment by fixation in a matrix followed by either amplicon sequencing or shotgun sequencing.
**Systems biology:**A holistic approach, often employing quantitative modeling, to study biological systems that cannot be reduced to the sum of the systems individual parts.
**Targeted RNA sequencing:**Sequencing of specific RNA molecules using probes complementing the transcript of interest.
**Transcriptome:**The sum of RNA transcripts produced by a single organism.
**Western blotting:**Separation and identification of proteins in a gel matrix using antibodies.

Historically, the phenotypic variation of plants and animals has been attributed to the interplay between **genomic** properties (Koonin et al., 2000) and environmental factors (Schmid, 1992). However, a long history of research on some insects and domestic vertebrates suggested that microorganisms associated with host animals should also be included in the equation. For example, termites have long been known (Leidy, 1881) to require gut microbes to be able to digest their food. In the last decade, researchers have benefited from the rapid development of high-throughput sequencing technology to more intensively explore how the **metagenomic** features of host-associated microorganisms also shape plant and animal phenotypes (Gilbert et al., 2018; Stringlis et al., 2018). These advances have expanded our knowledge on the role of host-microbe interactions in the evolution and ecology of modern-day organisms and how knowledge of such interactions can be beneficial in applied sciences. They basically revealed the termite example to be closer to the norm than the exception. Although individually both genomic and metagenomic approaches have proven useful for understanding many biological processes, each type of study has typically ignored the effect of the other domain and, critically, their interplay. Hence, the knowledge gained through such approaches is, at the very least, incomplete. The recognition of the importance of these host-microbiota interactions has recently opened up new research avenues based on the integrated analysis of coupled genomic and metagenomic data (Limborg et al., 2018), which can be referred to as the research field of **hologenomics** (Figure 1A).

**Figure 1:**
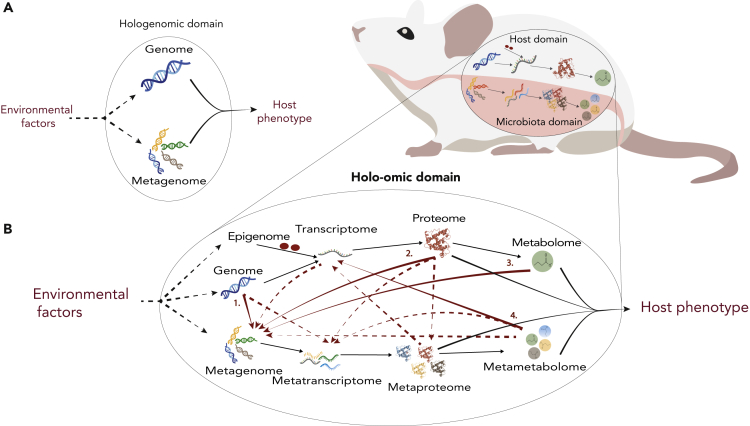
From Hologenomic to Holo-Omic (A) Simplified visualization of the hologenomic domain. (B) Host-microbiota interactions within the holo-omic domain here exemplified by zooming in on the luminal surface of the host intestine. Red arrows indicate host-microbiota holo-omic interactions. Solid red arrows indicate interactions supported in the primary literature (numbers refer to the publications listed in Table 1), whereas dashed red arrows indicate potential holo-omic interactions that, to the best of our knowledge, have not yet been documented. Solid black arrows indicate omic levels influencing host phenotype, and dashed black arrows indicate omic levels influenced by environmental factors.

Efforts to study the effects of host and microbial genes and their consequences have become embedded in layer upon layer of jargon. Because the concepts being discussed are new, some of these new terms are necessary, so as to have common reference points. But they only serve as effective reference points if they are well defined. Here we propose that hologenomics (the combined genetic content of the host and the **microbiota**) can be expanded to the **holo-omic** level by the incorporation of data from multiple **omic** levels from both host and microbiota domains (Limborg et al., 2018) (Figure 1B). This approach is inspired by elements originating from **systems biology** (e.g., metagenomics systems biology [Greenblum et al., 2012] and the use of **multi-omic** data integration [Bersanelli et al., 2016; Heintz-Buschart et al., 2016; Liu et al., 2020]). However, multi-omics implies omic data from only one domain, whereas holo-omics is defined by the incorporation of both host and microbial data. In theory, implementing a holo-omic approach would allow researchers to reveal a range of biomolecular interactions responsible for shaping the phenotype of complex organisms, using a variety of molecular tools, and would ultimately provide great potential for application across many different fields of research. The holo-omic toolbox requires both methodological and analytical tools. Within the methodological tools are the nucleic acid sequencing and mass spectrometry technologies that enable tracking the biomolecular pathways linking host and microbial genomic sequences with biomolecular phenotypes by generating (**meta**)**transcriptomes**, (**meta**)**proteomes**, and (**meta**)**metabolomes**. The same technologies also enable **epigenomic** and **exposomic** profiling, which can further contribute to disentangling the biochemical associations between host-microbiota-environment interactions and their effect on host phenotypes (Kumar et al., 2014; Rogler and Vavricka, 2015). The analytical tools required to extract useful information from the enormous amount of highly complex data generated by current high-throughput technologies are still limited. Association studies—identifying correlations between genetic variants and phenotypes—have been used to detect the genetic contributions to complex phenotypes (Welter et al., 2014). This approach has been extended to metabolomic profiles (Luo, 2015) and metagenomic variants (Blekhman et al., 2015; Qin et al., 2012), but methods that jointly leverage the multiple omic levels to infer the causal pathways between genomic processes and phenotypes are still scarce.

In this context, the technology to generate large amounts of data to be used in a holo-omic context is already available, but the analytical tools to reveal and identify host-microbiota interactions are still limited. As a consequence, only a handful of research groups worldwide have been able to effectively implement the holo-omic approach. To contribute to the development of this new field, in this perspective we first revisit the available evidence for the biological importance of host-microbiota interactions. Second, we present how the holo-omic toolbox can be used to study host-microbiota interactions at varying levels of complexity to guide researchers through applying the holo-omic approach. Third, we showcase the potential provided by the holo-omic approach to host-microbiota interactions in both basic and applied biological sciences and finally we identify the limiting factors that currently prevent the widespread implementation of the holo-omic approach and discuss possible solutions to overcome them.

## Host-Microbiota Interactions in Light of Holo-Omics

The holo-omic approach to host-microbiota interactions relies on three major assumptions: (1) host-associated microorganisms interact not only with each other but also with their host (Bredon et al., 2018; Fischer et al., 2017; Stringlis et al., 2018; Vaishnava et al., 2011); (2) these interactions affect, either positively or negatively, central biological processes of hosts and microorganisms (Wu and Wu, 2012); and (3) the interplay can be traced using biomolecular tools (Bansal et al., 2010; Bredon et al., 2018; Kelly et al., 2015; Virtue et al., 2019).

It has been estimated that the number of host-associated microbial cells and genes greatly outnumber that of their hosts' (Gilbert et al., 2018; Stringlis et al., 2018). These microorganisms do not passively inhabit the surfaces of their hosts but instead continuously interact with each other and their hosts through a myriad of complex feedback processes (e.g., Falcinelli et al., 2015; Kelly et al., 2015; Stringlis et al., 2018). For example, host genomic features are co-responsible for shaping the microbiota composition (Suzuki et al., 2019) through the differential biosynthesis of antibacterial peptides (Carvalho et al., 2012), differential composition of intestinal mucosa (Vaishnava et al., 2011), or differential release of nutrients (Reese et al., 2018). Gene expression interdependencies are also common between hosts and microorganisms. For instance, administration of *Lactobacillus rhamnosus* increases the uptake of fatty acids in zebrafish by down-regulating the transcription of host genes related to cholesterol and triglycerides metabolism (Falcinelli et al., 2015). Similarly, the metabolism of microbiota-derived butyrate in epithelial cells stabilizes the function of the hypoxia-inducible transcription factor, which regulates the expression of a number of genes related to host immunity (Kelly et al., 2015). Further examples of similar causal relationships between different omic levels from hosts and microorganisms are compiled in Table 1, and undoubtedly, many more will be revealed in the years to come.

**Table 1:** Examples of Holo-Omic Studies in the Current Litterature

Omic Levels	Organism	Major Findings	Reference	Arrow in Figure 1
Genome, microbial 16S	Mouse	20 host genes are associated with microbiome composition	Suzuki et al. (2019)	1
Genome, microbial 16S	Human	Genetic disposition for inflammatory bowel disease is associated with a reduction in abundance of the genus *Roseburia* in the gut microbiome	Imhann et al. (2018)	1
Transcriptome, metagenome	Pill-bug (*Armadillidium vulgare*)	Potential collaboration between microbiota and pill-bug in degrading lignocellulose	Bredon et al. (2018)	–
Proteome, microbial 16S	Mouse	Lack of the TLR5 protein increases Proteobacteria and decreases Bacteroidetes in microbiome and promotes gut inflammation	Carvalho et al. (2012)	2
Metabolome, metagenome	Thale cress (*Arabidopsis thaliana*)	Beneficial rhizobacteria induce excretion of the metabolite scopoletin that stimulates iron uptake and suppresses soil-borne pathogens	Stringlis et al. (2018)	3
Metametabolome, transcriptome	Human epithelial cells	Metabolism of microbiota-derived butyrate stabilizes the HIF transcription factor in human epithelial cells	Kelly et al. (2015)	4
Metametabolome, transcriptome	Human epithelial cells	The presence of microbiota-derived indole stimulates the expression of host genes connecting to the formation of tight junctions with a resulting higher pathogen resistance	Bansal et al. (2010)	4
Metametabolome, transcriptome	Mouse	Microbiota-derived indole controls expression of host *miR-181* expression that regulates adiposity and insulin sensitivity	Virtue et al. (2019)	4

Examples of studies considering different omic levels from hosts and associated microorganisms at different levels of resolution. When evidence of host-microbiota interactions are available numbers link the table to the corresponding interaction in Figure 1.

Host-microbiota interactions can have both positive and/or negative influences on host fitness. This has, for instance, been illustrated in studies on relatively well-defined bacteria-insect interactions. Such studies have revealed that the nature of these influences are often context dependent (Fry et al., 2004; Werren et al., 2008) and that these interactions can have both negative and positive influences on evolutionary adaptations (Bennett and Moran, 2015). For other, less studied and more complex host-microbiota consortiums, it has been found that positive interactions can, for instance, lead to increases in nutrient uptake through the degradation of recalcitrant organic compounds (Bredon et al., 2018), increase survival through modulating the resistance toward infectious diseases (Rosshart et al., 2017), or lengthen lifespan through modulating the aging process (Kim and Jazwinski, 2018). On the contrary, host-microbiota interactions can also have negative outcomes for the host. This is most obvious in the context of pathogens that cause infectious diseases (Fei and Zhao, 2013), but it is also apparent, for example, in the context of **dysbiosis** associated with chronic diseases such as inflammatory bowel syndrome (IBS) (Imhann et al., 2018). The origin of such microbial imbalances remains a cause of contention due to difficulty determining whether a disrupted microbiota is the cause or effect of a given illness (Walker, 2017) and it seems likely that such dysbioses have many different causes in different host species, genotypes, and contexts. This debate raises the question of how to determine what constitutes a healthy microbiome, a question that is difficult to answer, especially for wild organisms, owing to inter-population variation caused by environmental and genetic factors as well as the lack of functional annotation of many microbial genes (Lloyd-Price et al., 2016).

All these examples highlight the relevance of acknowledging and understanding the biomolecular interactions occurring between different omic levels of hosts and microorganisms. In the following section we will describe how holo-omics can be implemented by addressing different methodological, experimental, and analytical approaches.

## Implementing the Holo-Omic Approach

**Figure 2:**
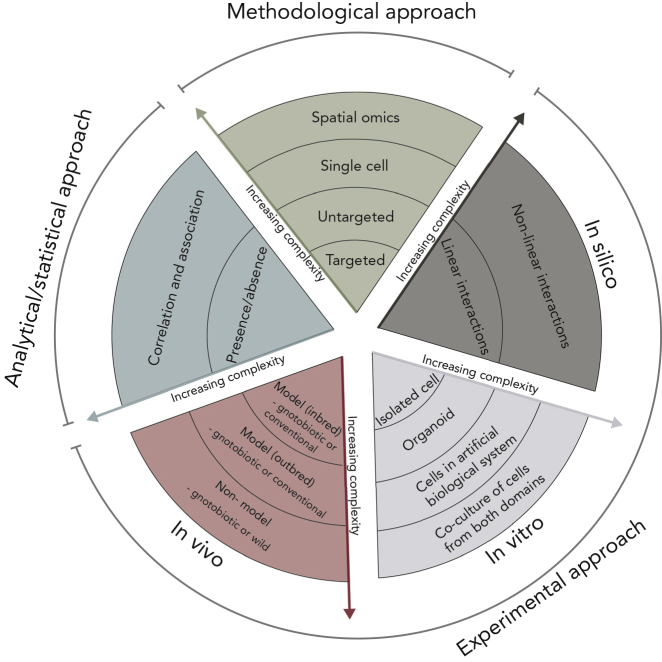
Overview of Different Approaches in Holo-Omics and Their Influence on the Level of Complexity Approaches are divided into methodological, experimental, and statistical. Arrows indicate the level of complexity relative to each segment of the figure.

The holo-omic approach can be implemented by using a range of different methodological tools in diverse experimental setups that might require a variety of analytical and statistical approaches (Figure 2). Regarding data generation, most studies linking the host and the microbiota domains have relied on targeted approaches (e.g., **amplicon sequencing**, **targeted RNA-sequencing**, and **western blotting**) to characterize the microbial domain. However, untargeted approaches (e.g., **shotgun DNA sequencing** and **shotgun proteomics**), which non-selectively provide a snapshot of nucleotides, proteins, and metabolites present in a sample, are progressively complementing or replacing targeted approaches. For instance, coupled untargeted host/microbe data from shotgun sequencing offers advantages over targeted approaches, such as the construction of metagenome assembled genomes (**MAGs**) from metagenomic data (Almeida et al., 2019) and the generation of individual genomic profiles (Blekhman et al., 2015). Furthermore, the (meta)genomic data needed for implementing the hologenomic approach to host-microbe interactions are often derived from samples containing DNA from both domains (Blekhman et al., 2015). At the same time, the ever-decreasing costs of sequencing coupled with increases in computational efficiency are expected to boost this trend toward shotgun sequencing (Quince et al., 2017). In recent years, **single cell sequencing** has expanded our ability to link specific genetic properties to single cells (Xu and Zhou, 2018), which could be used to study the interactions between *in vitro* cultures of eukaryotic and prokaryotic cells in great detail. In addition to this, the use of **spatial metagenomics** is capable of resolving the geographical distribution of individual microbes within a community (Sheth et al., 2019), and we foresee that this method will prove valuable in the future of holo-omics to highlight the effect of relative spatial orientation between host and microbial cells. In 10 years, incorporating a range of approaches in a single study with massive replication will probably be trivial from a cost perspective. In this context, the burden (and key challenge) is combining theoretical insight and analytical clarity.

If the metagenomic data include some proportion of host DNA, often considered as host contamination, *in silico* approaches can be used to also profile the host genotype and screen for potential associations between genetic markers and microbial traits (Blekhman et al., 2015). *In vitro* approaches, in which the host environment is reproduced in simpler physical models such as miniature organs grown from stem cells (i.e., epithelial organoids), might provide the required resolution when trying to uncover the interaction between well-defined binary interactions, e.g., the effect of microbiota-produced butyrate on host transcriptomics in epithelial cells (Kelly et al., 2015). *In vivo* approaches using single-symbiont or gnotobiotic organisms are chosen when trying to uncover the complete effect of a symbiont, beyond the effect of a single molecule (Koch and McFall-Ngai, 2019), whereas wild organisms might provide the most direct evidence about the effect of host-microbiota interactions in natural processes (Alberdi et al., 2016).

The implementation of a full holo-omic approach with multiple omic levels from both hosts and microorganisms begins with the generation of high-dimensional data. Depending on the aims of the study, data from each sample in the study can encompass measurements on genes, genomes, transcripts, proteins, or metabolites. Specifically, the microbiota can be characterized by hundreds of MAGs, thousands of gene orthologs, or millions of genes. The number of independent measurements and the high dimensionality of the resulting data pose significant challenges to traditional statistical approaches, such as correlation-based methods and linear models. One possible approach to reducing the complexity of the problem is to use some form of dimensionality reduction, such as clustering MAGs by taxonomy or ecological guilds (Zhao et al., 2018), or grouping genes by their functional properties (Qin et al., 2010). Although such dimensionality reduction simplifies the analyses and reduces computational complexity, it can lead to loss of biologically relevant information (Wang et al., 2019).

Pioneering studies in hologenomics have relied on association analyses to identify correlations between hosts and related microorganisms. Genome-wide association studies (**GWASs**) have linked specific loci in the host genome to the presence of pathogenic or beneficial microbes (Blekhman et al., 2015; Imhann et al., 2018). Similar approaches have been used in the study of epigenomes (Wan et al., 2016), metabolomes (Sekula et al., 2016), and proteomes (Okada et al., 2016). GWASs served as inspiration for metagenome-wide association studies (**MGWASs**) linking specific genes in the metagenome to phenotypic traits of interest in the host (Qin et al., 2012). So far, most methods used to integrate multi-omic data from both host and microbiota domains have relied on standard statistical methods, such as general linear models and linear mixed models in GWASs and MGWASs (Blekhman et al., 2015; Imhann et al., 2018; Qin et al., 2012). These methods are often hampered by the high-dimensional nature of the metagenomic data, highlighting the need for specialized methods to deal with highly complex holo-omic data (Wang et al., 2019).

Aiming to advance holo-omic research beyond association analyses, we recently introduced a methodological framework proposing a two-step approach to reveal the mechanisms underlying phenotypic variance modulated by the interactions between the host and related microorganisms (Limborg et al., 2018): an initial association phase based on GWAS and MGWAS analysis, followed by an interaction phase to identify bidirectional interactions at different omic levels. The initial association phase can identify variants (SNPs) within the genome and metagenome (e.g., amplicon sequence variants, operational taxonomic units, MAGs, or genes) associated with certain host phenotypes. In the following interaction phase, the effects of the associated GWAS variants on other omic domains are explored, thus identifying the important aspects of the molecular machinery that lead from genotypic variation to phenotypic variation. Although the two-step approach allows us to dig deeper into the interactions between the different omic domains that affect the phenotype, we are still limited by the power of the GWAS performed in the first step in identifying causal variants. In essence, the first step acts as a dimensionality reduction step, reducing the space of interactions that need to be interrogated. The problem of integrated inference by leveraging different omics data is a difficult one, and the development of computational methods in this field have been hindered by the inherent complexity of holo-omic data and the biological process underlying them. The current state of the art in integrating different omics dataset relies either on network-based methods (Langfelder and Horvath, 2008), regularized regression-based methods (Rohart et al., 2017), or other niche tools (Hernandez-Ferrer et al., 2017). However, none of these methods were designed for the analysis of metagenomic, metatranscriptomic, or metametabolomic data.

The methodological, experimental, and analytical approaches mentioned above are challenged by the high costs of data generation and the complexity of downstream analyses. This requires that researchers consider at least three fundamental questions about the system under study before taking on a holo-omic study (Box 2).

Box 2 **Three Main Questions that Researchers Need to Consider to Maximize the Outcome of a Holo-Omic Study**

(1) Are host-microbiota interactions relevant in the system under study?

Researchers must assess whether host-microbiota interactions are relevant for understanding the system under study. The impact of microorganisms associated with complex hosts is now regarded as almost universal (Barko et al., 2018), but the effect sizes can vary from low (Kong et al., 2019) to high (Rosshart et al., 2017) values. Hence, an initial screening of the variability of hosts' phenotypic traits and microbial communities associated with them is recommendable to elucidate potential correlations. This could be done using a cost-effective targeted gene sequencing approach to later study the system in more detail using non-targeted approaches.

(2) Is it meaningful to implement a holo-omic approach?

**Figure 3:**
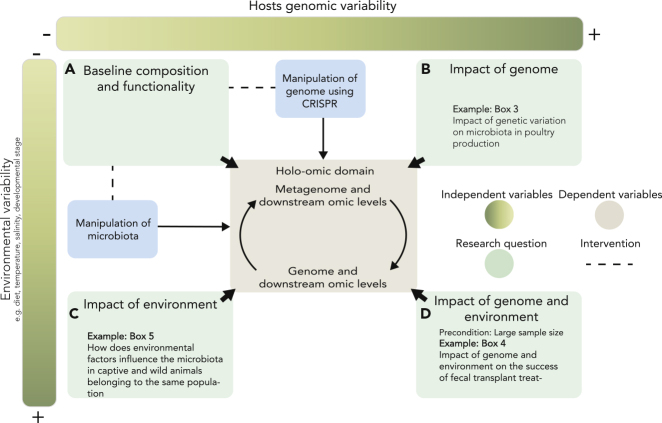
Overview of Different Variables that Will Impact Holo-Omic Studies In this conceptualization, two independent variables, the environment and the host genome, affect dependent variables (center), the metagenome, and downstream omic levels and their interactions with the host genome and derived omic levels. Different combinations enable implementing different types of experimental approaches. (A) When both genetic background and environment are constant (e.g., laboratory conditions) the underlying composition and functionality of the microbiota as well as the underlying interaction with the host domain can be determined. These conditions allow researchers to manipulate microbiota composition and functionality and to manipulate the host genome (e.g., using CRISPR-Cas9 genome editing technology). (B) When the genetic background is variable and the environment is relatively consistent, the impact of genetic variants on downstream omic levels can be isolated. (C) When the genetic background is similar and the environment is variable, the impact of environmental factors on the different omic levels can be studied. (D) When both genetic background and environment are variable, the high level of variability will complicate the isolation of factors responsible for modifying the omic levels. Increasing sample size can mitigate this problem.

It is necessary to evaluate whether the implementation of a holo-omic approach is reasonable given the properties of the biological system and its environment. Holo-omics relies on the premise that genomic and metagenomic differences across individuals, treatments, populations, or species affect biological processes and phenotypic outcomes. Thus, the existence of genomic or metagenomic variation in the system is essential. It is also necessary to bear in mind that the capacity to recover genomic and metagenomic signatures is largely affected by environmental variables (Figure 3). The background noise introduced by these variables contains information on how the environment influences the dependent variables (Figure 3), but as they are often difficult to measure or control in non-laboratory settings they will often complicate signal recovery. Factors extrinsic to the host (diet, temperature, humidity, etc.) are known to affect both the composition of the microbiota and the expression of its genes (Cernava et al., 2019; David et al., 2014; Moran and Yun, 2015). The level and structure of (meta)genomic and environmental variation will therefore dictate the biological meaning and design of any holo-omic study (Figure 3).

Assessing the economic and technical feasibility of the study is also paramount. This includes acknowledging the genome size of the host, as the genome of some species can be magnitudes larger than others, e.g., amphibians (Nowoshilow et al., 2018) versus birds (Zhang et al., 2014), or questioning whether optimal sample preservation conditions can be ensured, especially critical for (meta)transcriptomics (Ferreira et al., 2018). Assessing the biomolecular properties of the samples (e.g., host:microbiota DNA/RNA ratios) is a relevant preliminary step that aids in outlining an optimal study design (Human Microbiome Project Consortium, 2012).

(3) Which omic levels are relevant and how to maximize the amount of useful data derived from them?

Lastly, researchers should identify which omic levels are the most relevant, both by considering the biological and technical features of the experimental system and their relevance to the research questions. The omic levels selected for analysis will largely determine the number of samples to include (Ching et al., 2014; Hong and Park, 2012), where and how to collect the samples (e.g., which part of the intestinal tract (Kokou et al., 2019), preservation and storage conditions (Ferreira et al., 2018; Hickl et al., 2019), sequencing depth, or how to maximize the amount of biological information coming from them (Quince et al., 2017). The ability of the downstream statistical analyses is dependent not just on these factors but also on the genetic architecture of the phenotype being studied. Prior knowledge of the functional basis of the phenotype can be used to markedly improve the experimental design and improve the power of the statistical analyses (Kichaev et al., 2019).

## Applying the Holo-Omic Approach across Biological Sciences

The holo-omic approach outlined above can be implemented in many basic and applied biological research fields to address relevant scientific questions concerning host-microbiota interactions. In the following, we showcase and discuss the application of the holo-omic approach in diverse fields of biological sciences. For some fields, we include boxes containing case examples to better illustrate its potential implementation.

### Agricultural and Aquacultural Sciences

The holo-omic approach could prove a meaningful tool in developing animal and plant production as microorganisms are increasingly considered essential assets to improve efficiency and sustainability (Małyska et al., 2019). Among other strategies, animal feed and feed additives are used to modulate animal gut microbiota and improve host growth and health. More sustainable feed formulas are being developed such as the use of seaweed to decrease dairy cows methane emissions (Machado et al., 2014). It has also been suggested that piscivorous fish can be fed with a plant-based diet in aquaculture systems to replace fish meal (Gatlin et al., 2007). On the other hand, feed additives, such as probiotics, prebiotics, and synbiotics, are extensively used in animal breeding owing to many attributed benefits including protection against pathogens, stimulation of the immunological response, and increment of production capacity, but yet, little is known about their specific mode of action (Markowiak and Śliżewska, 2018). For instance, positive effects were reported on the use of probiotics to control diarrhea syndrome in post-weaning piglets (Kyriakis et al., 1999) and have been found to result in decreased mortality in rainbow trout (Irianto and Austin, 2002). The implementation of the holo-omic approach can help us unveil how feed, microbiota, and the host interact in the intestinal environment, which could prove essential for optimizing the production of host organisms and improving management practices (Box 3). A similar initiative implemented for plants could aim at enhancing adaptation and response to rapid climate change.

Box 3 **Implementing the Holo-Omic Approach in Poultry Farming**

Chickens are an important source of high-quality protein for a large proportion of the human population. The gut microbiota of broilers (chicken bred for meat production) is highly variable since they are slaughtered before reaching an age in which the microbial community dynamics stabilize (Rychlik, 2020). Although the administration of probiotics and prebiotics to modulate the gut microbiota is becoming increasingly popular, results are still inconclusive and further research is needed (Ducatelle et al., 2015).

(1) Are host-microbiota interactions relevant in the system under study?

The controlled environmental conditions in intensive poultry production systems, which use the same feeding strategy and environment for all individuals, indicate that the likely reason behind variation in the chicken performance and their gut microbiota when administered pre- and probiotics might be explained by microbial founding effects and microgenomic variation of broilers (Box 2. Figure 3B: Impact of Genome) not only across but also within breeds. An initial examination of the genotypes along with a targeted screening of the microbiota of each individual in the broiler population can allow researchers to discover any potential association between the two domains using GWASs and MGWASs with a particular focus on pre- and probiotics-related phenotypic responses (e.g., inflammatory markers, stress response molecules).

(2) Is it meaningful to implement a holo-omic approach?

Pre- and probiotics interact with native gut microorganisms as well as with the host. The gut microbiota of broilers is relatively simple because of the closed environment where the broilers are reared. The genetic diversity of conventionally bred broilers is low, yet even small interindividual differences can be crucial and might have wide implications on the response to pre- and probiotics. These system properties allow the successful application of the holo-omic framework for obtaining relevant microbe-microbe and microbe-host interactions, which can help researchers optimize feed additives design, production, and administration, thereby preventing production inefficiency driven by gut dysbiosis.

(3) Which omic levels are relevant and how to maximize the amount of useful data derived from them?

If associations are detected between the (meta)genome and host phenotypic traits, the study of transcriptome, metatranscriptome, and metabolome can unveil the nature of microbe-microbe and host-microbe interactions and how they affect the host. Detecting molecular pathways that are activated or deactivated in the presence of pre- and probiotics can enable researchers to identify production-related phenotypic changes in the host.

### Biotechnology

The holo-omic approach could also contribute to developing and optimizing biotechnological solutions. For instance, it could be used to better understand host-microbiota systems capable of enzymatically degrading complex polysaccharides (Ni and Tokuda, 2013) in the search for novel sustainable ways of transforming organic waste compounds into industrially relevant biomolecules and biofuels. Many wood-feeding organisms are capable of partially digesting lignocellulose into glucose, but to complete the degradation they need the complementary enzymes produced by their resident microbes (Bredon et al., 2018). Similarly, several studies based on metagenomics and metaproteomics in termites have shown that the microbiota is responsible for producing some of the most complex enzymes involved in the degradation of lignocellulose (Ni and Tokuda, 2013). Most of such complex biochemical reactions occur under anaerobic environments; hence, setting up appropriate bioreactors tends to be a complex process. The holo-omic approach can assist in determining specific bioreactor conditions by ascertaining the enzymatic and metabolic contribution of microorganisms and animal hosts, thus facilitating the replication of the optimal chemical conditions that mimic the hosts' gut environment (Gutleben et al., 2018).

### Biomedical Research

Incorporating the holo-omics approach to biomedical research offers an exciting new avenue toward better treatments of many modern human diseases. Most people in industrialized societies exhibit depauperate gut microbiotas (Gupta et al., 2017), which is often held co-responsible for the concomitant explosion in the rate of autoimmune diseases (Bach, 2002), all diseases that have been associated with a dysbiotic microbiome in patients including IBS (Imhann et al., 2018), diabetes, or colorectal cancer (Feng et al., 2015). Although we rarely know whether such a dysbiotic microbiome (Walker, 2017) is the cause or an effect of a disease trait, it is now clear that the field of holo-omics provides an attractive approach to better understand how such changes in host-microbiome interactions occur and potentially how they can be reverted to healthy states. Better understanding of how human genotypes and the exposome of an individual affect the interactions between patients and associated microorganisms would enable advances toward more accurate personalized medicine (Ginsburg and McCarthy, 2001). A holo-omics-based personalized medicine would recognize not only the genetic and exposomic features of patients but also the associated microbiota (Box 4).

Box 4 **Holo-Omic Approach to Fecal Transplant Treatments**

The use of fecal transplants, i.e., transferring fecal material from a healthy donor to a patient with a gastrointestinal disorder, is now becoming a promising treatment for multiple gastrointestinal disorders (Mcilroy et al., 2019). Although such treatments have shown some success, outcomes often vary among patients despite receiving the same treatment (Sbahi and Di Palma, 2016). Therefore, we hypothesize that the holo-omics approach can be applied to improve the success rate of such treatments by matching the genotype between fecal donors and recipients similar to procedures for organ transplants.

(1) Are host-microbiota interactions relevant in the system under study?

The success of fecal transplants relies on the capability of beneficial microbes from the donor fecal sample being able to colonize and establish themselves in the gut environment of the recipient. One can hypothesize that the probability of success relates to differences among patient gut “environments” that depends not only on the existing microorganism community but also on the genotype or **epigenotype** of the human host (Box 2. Figure 3D: Impact of Genome and Environment).

(2) Is it meaningful to implement a holo-omic approach?

The information gained from a holo-omic approach will ultimately lead to more efficient treatments by, for example, optimizing the biological match between a fecal donor and recipient. For example, a holo-omics analysis in a controlled cohort can reveal concrete genotypes of a host that are associated with the gut microbiota composition. Then, once we have accumulated knowledge about specific candidate genes directly associated with composition and function of gut microbiota, we can screen these genes to optimize the genetic match between donor and recipient, thereby improving the odds that the recipient is likely to adopt the healthy microbes from the donor and thereby counteract the negative effect from microbes such as *Clostridioides difficile* (Gough et al., 2011).

(3) Which omic levels are relevant and how to maximize the amount of useful data derived from them?

A holo-omic approach to identify the factors underlying the differential success of fecal transplants could include the patients' genomic and epigenomic features coupled with transcriptomic, metagenomic, metatranscriptomic, and metabolomic variation before and after a fecal transplant. Associating these features with the success of the treatment, and with each other, would shed light on the functional changes introduced by the transplant, which would enable identifying the factors leading to a success or failure of the treatment.

### Ecology and Evolution

Implementation of a holo-omic approach holds the potential to address many basic questions regarding the ecology and evolution of species. Most pertinent of these are in regards to the holobiont and testing specific hypotheses derived from the **hologenome theory of evolution** (Rosenberg et al., 2007). For example, how does selection occur on the holobiont and what mechanisms underpin the cross talk between the host and microbiota axes. One potential application is to measure the impact of microorganisms in vertebrate adaptation and improve predictions from anthropogenic disturbances, such as climate change and habitat destruction, on species distributions. It has been proposed that metagenomes could confer enhanced adaptive capacity to their hosts (Alberdi et al., 2016), potentially enabling rapid adaptation to changing environmental conditions (Fontaine and Kohl, 2020). The adaptive capacity of hosts and their associated microbiota through linking specific host genotypes with metagenomes has been demonstrated with regard to toxicity resilience (Macke et al., 2017), heat tolerance (Moran and Yun, 2015), drought and desiccation (Cernava et al., 2019), disease resistance (Rosshart et al., 2017), and nutrient acquisition (Falcinelli et al., 2015). Through characterizing host-microbiota pathways it is possible to catalog these interactions and begin to assess evolutionary adaptations within the metagenome. This could enable metagenomic—rather than only genomic (e.g., Razgour et al. (2019))—adaptations to be considered when predicting species range shifts owing to climate change and potentially improves the predictive capacity of species distributions. Likewise, such an approach could also be adopted to improve predictions of the adaptive capacity relevant to modeling invasive species (Fontaine and Kohl, 2020), enabling better estimates of invasion trajectories and ecological impact forecasts.

### Species Conservation

Holo-omics could also be relevant for developing optimal active conservation actions, such as captivity breeding and animal translocations (Box 5). As captive conditions differ extensively from those experienced in the wild, many species kept in captivity diverge in their microbiota compositions compared with their wild counterparts (McKenzie et al., 2017). This could have implications for attempts to translocate species (i.e., introduction, re-introduction, and re-stocking) as the functionality of the microbiota might be compromised thereby diminishing the chance of successful translocation (Bahrndorff et al., 2016). Microbiota composition and functionality varies between local environments, and identifying the local variants can impact conservation effort success. Although conservationists have traditionally focused on a species genetic traits (Allendorf et al., 2010), the holo-omic approach posit to match this information with information on microbiota composition and functionality, to avoid mixing populations with different hologenomic adaptations to a given environment. Matching captive individuals with a “wild microbiota” prior to their release and monitoring their fitness and associated temporal changes of the microbiota in the wild could reveal the efficiency of the holo-omic approach in the field of conservation.

Box 5 **Implementing the Holo-Omic Approach in Conservation Biology**

In winter, the Western capercaillie (*Tetrao urogallus*) feeds almost exclusively on conifer needles rich in resin and phenol and low in nutrients (Bryant, 1980). It has been proposed that the microbiota might be of major importance in aiding the metabolism of these hard-to-digest compounds (Wienemann et al., 2011). Failure of translocated captive-bred individuals to survive in the wild is suspected to be a consequence of the lack of specific microbes capable of digesting the toxic compounds in the diet (Wienemann et al., 2011).

(1) Are host-microbiota interactions relevant in the system under study?

The highly specialized diet with many hard-to-digest components of the capercaillie suggests that the digestion of these compounds might be facilitated by the microbiota. An initial screening using shallow shotgun sequencing will indicate microbial differences between wild and captive capercaillies to identify taxa and functions related to the degradation of resin and phenol that might be missing in captive individuals.

(2) Is it meaningful to implement a holo-omic approach?

If the captive bred individuals originate from the same population as they are meant to be released in, then the system is relatively simple with two similar genetic backgrounds (wild and captive-bred from the same wild population). This means that the effect of genetics is roughly the same for wild and captive conspecifics, which will allow researchers to study the impact of the environment (i.e., a diet of pine needles) on microbiota functionality (Box 2. Figure 3C: Impact of Environment). If captive bred individuals originating from one population are to be released to increase the number of animals in another population, then it becomes increasingly important to consider that host gene functionality between populations might vary and the contribution from the microbiota to these functions are likely to also vary. It is therefore important to consider if the genes or allelic variants necessary for an optimal digestion of conifer needles are present, either inherent to the host genome or in the metagenome.

(3) Which omic levels are relevant and how to maximize the amount of useful data derived from them?

If the initial screening of the metagenome indicates a lack of functions related to the metabolism of phenol and resin in captive capercaillies, the next step will be to gradually feed them more of their natural diet of pine needles and subsequently screen both the metagenome and (meta)transcriptome. Screening both the transcriptome and metatranscriptome will allow conservationists to uncover complementary interactions between host and microbiota genes. If the genes of interest are suddenly present and expressed then the dietary change has been enough to provide the captive capercaillies with a “wild microbiota” and released animals can then be monitored and their fitness compared with control animals with a captive microbiota. If captive individuals fail to acquire the needed functionalities through the gradual change to a more natural diet other vectors of enrichment should be tested (e.g., natural soil or feces from wild capercaillies).

## Current Limitations and Future Perspectives

Although holo-omics represent a valuable tool for many fields, its implementation is still hampered by economic, technical and biological limitations. A main economic hurdle is the high cost of shotgun sequencing. Targeted sequencing or DNA microarrays approaches can be cost-effective alternatives for characterizing (meta)genomes in some cases, although shallow shotgun sequencing can in some instances recover higher taxonomic resolution at the same cost, while also providing direct inference about functionality (Hillmann et al., 2018). Targeted approaches might enable researchers to establish correlation between the presence of specific microorganisms and genetic or phenotypic traits of the host, but to infer causation the use of shotgun sequencing will often be necessary to provide whole genome resolution. Alternatively, a cost-effective approach, mostly useful when the microbial diversity is limited, is to combine targeted amplicon sequencing with deep shotgun sequencing on a subset of samples in a dataset (Lesker et al., 2020). However, if the required resolution can only be achieved using shotgun approaches, it is essential to consider the costs of generating the required amount of data and to design the experiments and sampling strategies accordingly. One of the advantages of the holo-omic approach is that all generated data are useful in qualitative terms (i.e., host DNA is valuable information, rather than contamination). However, this does not imply that all generated data are quantitatively useful. The usefulness and cost-effectiveness are influenced by the proportion of host- or microbiota-derived nucleic acids, amino acids, or metabolites. These proportions change drastically across sample types (Marotz et al., 2018) and host taxa (Human Microbiome Project Consortium, 2012; Singh et al., 2014), and an incorrect estimation can require drastic budget adjustments.

The holo-omic approach faces essential challenges, such as those linked to the quantity and complexity of the data to be analyzed. The interactions between different microbes, each synthesizing and metabolizing a variety of molecules, and the interactions between microbes and host cells is extremely complex, with the nature of these interactions being far from uniform and linear. This demands an integrative approach that can account for the different data types under the same inference framework. Generative/mechanistic models exist for many of the individual omics data, such as transcriptome, proteome, and metagenome, but integrating these models under a single inference framework is challenging, given the different data types (compositional versus absolute abundance, discrete vs. continuous) and the vastly different biological processes that underlie them. Thus, developing mechanistic models for such data are an active area of research. In addition, in most current studies, the holo-omics data contain a lot of missing values, e.g., the transcriptomics and microbiome data may not come from the same individuals, and the generated data fall under the small N (sample size), large P (features) paradigm. That is, the data contain a limited number of independent observations of a large number of features. In the case of holo-omics data, features can include millions of genomic variants, mRNA quantification for thousands of genes, abundance estimates of hundreds to thousands of taxa in the microbiome, and tens to hundreds of phenotypes such as health parameters and growth rates. Unfortunately, the large number of features (P) are not accompanied by a corresponding increase in sample sizes (N), owing to the high cost of generating such comprehensive data for a large number of individuals. Identifying the important determining features in such datasets can be very challenging given the limited number of independent observations. Statistical advances in the last decade including development of deep learning methods are helping address the challenges posed by the high dimensionality and complex correlation structure of the data. Development of such methods is an area of active research where several advances have been made in integrating host-microbiome data (Bersanelli et al., 2016; Heintz-Buschart et al., 2016; Liu et al., 2020).

## Conclusion

Although still challenged by many limitations, the feasibility to conduct holo-omic research will only increase in the near future, aided by the continuous publication and improvement of macro- and microorganism genomes, the decrease of costs for DNA/RNA sequencing and mass spectrometry, the increase of computational capacities, and the uninterrupted development of analytical tools to analyze the huge amounts of data generated. These trends will allow a broader range of research groups to conduct holo-omic studies and as the need for detailed information on host-microbiota interactions increases in both applied and basic sciences there is no doubt that the holo-omic approach will gain popularity in the future.

## References

[bib1] Alberdi A., Aizpurua O., Bohmann K., Zepeda-Mendoza M.L., Gilbert M.T.P. (2016). Do vertebrate gut metagenomes confer rapid ecological adaptation?. Trends Ecol. Evol..

[bib2] Allendorf F.W., Hohenlohe P.A., Luikart G. (2010). Genomics and the future of conservation genetics. Nat. Rev. Genet..

[bib3] Almeida A., Mitchell A.L., Boland M., Forster S.C., Gloor G.B., Tarkowska A., Lawley T.D., Finn R.D. (2019). A new genomic blueprint of the human gut microbiota. Nature.

[bib4] Bach J.-F. (2002). The effect of infections on susceptibility to autoimmune and allergic diseases. N. Engl. J. Med..

[bib5] Bahrndorff S., Alemu T., Alemneh T., Lund Nielsen J. (2016). The microbiome of animals: implications for conservation biology. Int. J. Genomics Proteomics.

[bib6] Bansal T., Alaniz R.C., Wood T.K., Jayaraman A. (2010). The bacterial signal indole increases epithelial-cell tight-junction resistance and attenuates indicators of inflammation. Proc. Natl. Acad. Sci. U S A.

[bib7] Barko P.C., McMichael M.A., Swanson K.S., Williams D.A. (2018). The gastrointestinal microbiome: a review. J. Vet. Intern. Med..

[bib8] Bennett G.M., Moran N.A. (2015). Heritable symbiosis: the advantages and perils of an evolutionary rabbit hole. Proc. Natl. Acad. Sci. U S A.

[bib9] Bersanelli M., Mosca E., Remondini D., Giampieri E., Sala C., Castellani G., Milanesi L. (2016). Methods for the integration of multi-omics data: mathematical aspects. BMC Bioinformatics.

[bib10] Blekhman R., Goodrich J.K., Huang K., Sun Q., Bukowski R., Bell J.T., Spector T.D., Keinan A., Ley R.E., Gevers D. (2015). Host genetic variation impacts microbiome composition across human body sites. Genome Biol..

[bib11] Bredon M., Dittmer J., Noël C., Moumen B., Bouchon D. (2018). Lignocellulose degradation at the holobiont level: teamwork in a keystone soil invertebrate. Microbiome.

[bib12] Bryant J.P. (1980). Selection of winter forage by subarctic browsing vertebrates: the role of plant chemistry. Annu. Rev. Ecol. Syst..

[bib13] Carvalho F.A., Koren O., Goodrich J.K., Johansson M.E., Nalbantoglu I., Aitken J.D., Su Y., Chassaing B., Walters W.A., González A. (2012). Transient inability to manage proteobacteria promotes chronic gut inflammation in TLR5-deficient mice. Cell Host Microbe.

[bib14] Cernava T., Aschenbrenner I.A., Soh J., Sensen C.W., Grube M., Berg G. (2019). Plasticity of a holobiont: desiccation induces fasting-like metabolism within the lichen microbiota. ISME J..

[bib15] Ching T., Huang S., Garmire L.X. (2014). Power analysis and sample size estimation for RNA-Seq differential expression. RNA.

[bib16] David L.A., Maurice C.F., Carmody R.N., Gootenberg D.B., Button J.E., Wolfe B.E., Ling A.V., Devlin A.S., Varma Y., Fischbach M.A. (2014). Diet rapidly and reproducibly alters the human gut microbiome. Nature.

[bib17] Ducatelle R., Eeckhaut V., Haesebrouck F., Van Immerseel F. (2015). A review on prebiotics and probiotics for the control of dysbiosis: present status and future perspectives. Animal.

[bib18] Falcinelli S., Picchietti S., Rodiles A., Cossignani L., Merrifield D.L., Taddei A.R., Maradonna F., Olivotto I., Gioacchini G., Carnevali O. (2015). Lactobacillus rhamnosus lowers zebrafish lipid content by changing gut microbiota and host transcription of genes involved in lipid metabolism. Sci. Rep..

[bib19] Fei N., Zhao L. (2013). An opportunistic pathogen isolated from the gut of an obese human causes obesity in germfree mice. ISME J..

[bib20] Feng Q., Liang S., Jia H., Stadlmayr A., Tang L., Lan Z., Zhang D., Xia H., Xu X., Jie Z. (2015). Gut microbiome development along the colorectal adenoma-carcinoma sequence. Nat. Commun..

[bib21] Ferreira P.G., Muñoz-Aguirre M., Reverter F., Sá Godinho C.P., Sousa A., Amadoz A., Sodaei R., Hidalgo M.R., Pervouchine D., Carbonell-Caballero J. (2018). The effects of death and post-mortem cold ischemia on human tissue transcriptomes. Nat. Commun..

[bib22] Fischer C.N., Trautman E.P., Crawford J.M., Stabb E.V., Handelsman J., Broderick N.A. (2017). Metabolite exchange between microbiome members produces compounds that influence Drosophila behavior. Elife.

[bib23] Fontaine S.S., Kohl K.D. (2020). The gut microbiota of invasive bullfrog tadpoles responds more rapidly to temperature than a non-invasive congener. Mol. Ecol..

[bib24] Fry A.J., Palmer M.R., Rand D.M. (2004). Variable fitness effects of Wolbachia infection in *Drosophila melanogaster*. Heredity.

[bib25] Gatlin D.M., Barrows F.T., Brown P., Dabrowski K., Gaylord T.G., Hardy R.W., Herman E., Hu G., Krogdahl Å., Nelson R. (2007). Expanding the utilization of sustainable plant products in aquafeeds: a review. Aquac. Res..

[bib26] Gilbert J.A., Blaser M.J., Caporaso J.G., Jansson J.K., Lynch S.V., Knight R. (2018). Current understanding of the human microbiome. Nat. Med..

[bib27] Ginsburg G.S., McCarthy J.J. (2001). Personalized medicine: revolutionizing drug discovery and patient care. Trends Biotechnol..

[bib28] Gough E., Shaikh H., Manges A.R. (2011). Systematic review of intestinal microbiota transplantation (fecal bacteriotherapy) for recurrent *Clostridium difficile* infection. Clin. Infect. Dis..

[bib29] Greenblum S., Turnbaugh P.J., Borenstein E. (2012). Metagenomic systems biology of the human gut microbiome reveals topological shifts associated with obesity and inflammatory bowel disease. Proc. Natl. Acad. Sci. U. S. A..

[bib30] Gupta V.K., Paul S., Dutta C. (2017). Geography, ethnicity or subsistence-specific variations in human microbiome composition and diversity. Front. Microbiol..

[bib31] Gutleben J., Chaib De Mares M., van Elsas J.D., Smidt H., Overmann J., Sipkema D. (2018). The multi-omics promise in context: from sequence to microbial isolate. Crit. Rev. Microbiol..

[bib32] Heintz-Buschart A., May P., Laczny C.C., Lebrun L.A., Bellora C., Krishna A., Wampach L., Schneider J.G., Hogan A., de Beaufort C. (2016). Integrated multi-omics of the human gut microbiome in a case study of familial type 1 diabetes. Nat. Microbiol..

[bib33] Hernandez-Ferrer C., Ruiz-Arenas C., Beltran-Gomila A., González J.R. (2017). MultiDataSet: an R package for encapsulating multiple data sets with application to omic data integration. BMC Bioinformatics.

[bib34] Hickl O., Heintz-Buschart A., Trautwein-Schult A., Hercog R., Bork P., Wilmes P., Becher D. (2019). Sample preservation and storage significantly impact taxonomic and functional profiles in metaproteomics studies of the human gut microbiome. Microorganisms.

[bib35] Hillmann B., Al-Ghalith G.A., Shields-Cutler R.R., Zhu Q., Gohl D.M., Beckman K.B., Knight R., Knights D. (2018). Evaluating the information content of shallow shotgun metagenomics. mSystems.

[bib36] Hong E.P., Park J.W. (2012). Sample size and statistical power calculation in genetic association studies. Genomics Inform..

[bib37] Human Microbiome Project Consortium (2012). A framework for human microbiome research. Nature.

[bib38] Imhann F., Vich Vila A., Bonder M.J., Fu J., Gevers D., Visschedijk M.C., Spekhorst L.M., Alberts R., Franke L., van Dullemen H.M. (2018). Interplay of host genetics and gut microbiota underlying the onset and clinical presentation of inflammatory bowel disease. Gut.

[bib39] Irianto A., Austin B. (2002). Use of probiotics to control furunculosis in rainbow trout, *Oncorhynchus mykiss* (Walbaum). J. Fish Dis..

[bib40] Kelly C.J., Zheng L., Campbell E.L., Saeedi B., Scholz C.C., Bayless A.J., Wilson K.E., Glover L.E., Kominsky D.J., Magnuson A. (2015). Crosstalk between microbiota-derived short-chain fatty acids and intestinal epithelial HIF augments tissue barrier function. Cell Host Microbe.

[bib41] Kichaev G., Bhatia G., Loh P.-R., Gazal S., Burch K., Freund M.K., Schoech A., Pasaniuc B., Price A.L. (2019). Leveraging polygenic functional enrichment to improve GWAS power. Am. J. Hum. Genet..

[bib98] Kim S., Jazwinski S.M. (2018). The gut microbiota and healthy aging. Gerontology.

[bib42] Koch E.J., McFall-Ngai M. (2019). Model systems for the study of how symbiotic associations between animals and extracellular bacterial partners are established and maintained. Drug Discov. Today Dis. Models.

[bib43] Kokou F., Sasson G., Friedman J., Eyal S., Ovadia O., Harpaz S., Cnaani A., Mizrahi I. (2019). Core gut microbial communities are maintained by beneficial interactions and strain variability in fish. Nat. Microbiol..

[bib44] Kong H.G., Kim H.H., Chung J.H., Jun J., Lee S., Kim H.M., Jeon S., Park S.G., Bhak J., Ryu C.M. (2019). The Galleria mellonella hologenome supports microbiota-independent metabolism of long-chain hydrocarbon beeswax. Cell Rep..

[bib45] Koonin E.V., Aravind L., Kondrashov A.S. (2000). The impact of comparative genomics on our understanding of evolution. Cell.

[bib46] Kumar H., Lund R., Laiho A., Lundelin K., Ley R.E., Isolauri E., Salminen S. (2014). Gut microbiota as an epigenetic regulator: pilot study based on whole-genome methylation analysis. MBio.

[bib47] Kyriakis S.C., Tsiloyiannis V.K., Vlemmas J., Sarris K., Tsinas A.C., Alexopoulos C., Jansegers L. (1999). The effect of probiotic LSP 122 on the control of post-weaning diarrhoea syndrome of piglets. Res. Vet. Sci..

[bib48] Langfelder P., Horvath S. (2008). WGCNA: an R package for weighted correlation network analysis. BMC Bioinformatics.

[bib49] Leidy J. (1881). Parasites of the Termites.

[bib50] Lesker T.R., Durairaj A.C., Gálvez E.J.C., Lagkouvardos I., Baines J.F., Clavel T., Sczyrba A., McHardy A.C., Strowig T. (2020). An integrated metagenome catalog reveals new insights into the murine gut microbiome. Cell Rep..

[bib51] Liang S., Wu X., Jin F. (2018). Gut-brain psychology: rethinking psychology from the microbiota-gut-brain axis. Front. Integr. Neurosci..

[bib52] Limborg M.T., Alberdi A., Kodama M., Roggenbuck M., Kristiansen K., Gilbert M.T.P. (2018). Applied hologenomics: feasibility and potential in aquaculture. Trends Biotechnol..

[bib53] Liu Z., Ma A., Mathé E., Merling M., Ma Q., Liu B. (2020). Network analyses in microbiome based on high-throughput multi-omics data. Brief. Bioinform..

[bib54] Lloyd-Price J., Abu-Ali G., Huttenhower C. (2016). The healthy human microbiome. Genome Med..

[bib55] Luo J. (2015). Metabolite-based genome-wide association studies in plants. Curr. Opin. Plant Biol..

[bib56] Machado L., Magnusson M., Paul N.A., de Nys R., Tomkins N. (2014). Effects of marine and freshwater macroalgae on in vitro total gas and methane production. PLoS One.

[bib57] Macke E., Callens M., De Meester L., Decaestecker E. (2017). Host-genotype dependent gut microbiota drives zooplankton tolerance to toxic cyanobacteria. Nat. Commun..

[bib58] Małyska A., Markakis M.N., Pereira C.F., Cornelissen M. (2019). The microbiome: a life science opportunity for our society and our planet. Trends Biotechnol..

[bib59] Markowiak P., Śliżewska K. (2018). The role of probiotics, prebiotics and synbiotics in animal nutrition. Gut Pathog..

[bib60] Marotz C.A., Sanders J.G., Zuniga C., Zaramela L.S., Knight R., Zengler K. (2018). Improving saliva shotgun metagenomics by chemical host DNA depletion. Microbiome.

[bib61] Mcilroy J.R., Segal J.P., Mullish B.H., Nabil Quraishi M., Gasbarrini A., Cammarota G., Ianiro G. (2019). Current and future targets for faecal microbiota transplantation. Hum. Microbiome J..

[bib62] McKenzie V.J., Song S.J., Delsuc F., Prest T.L., Oliverio A.M., Korpita T.M., Alexiev A., Amato K.R., Metcalf J.L., Kowalewski M. (2017). The effects of captivity on the mammalian gut microbiome. Integr. Comp. Biol..

[bib63] Moran N.A., Yun Y. (2015). Experimental replacement of an obligate insect symbiont. Proc. Natl. Acad. Sci. U S A.

[bib64] Nicholson J.K., Holmes E., Kinross J., Burcelin R., Gibson G., Jia W., Pettersson S. (2012). Host-gut microbiota metabolic interactions. Science.

[bib65] Ni J., Tokuda G. (2013). Lignocellulose-degrading enzymes from termites and their symbiotic microbiota. Biotechnol. Adv..

[bib66] Nowoshilow S., Schloissnig S., Fei J.F., Dahl A., Pang A.W.C., Pippel M., Winkler S., Hastie A.R., Young G., Roscito J.G. (2018). The axolotl genome and the evolution of key tissue formation regulators. Nature.

[bib67] Okada H., Ebhardt H.A., Vonesch S.C., Aebersold R., Hafen E. (2016). Proteome-wide association studies identify biochemical modules associated with a wing-size phenotype in *Drosophila melanogaster*. Nat. Commun..

[bib68] Qin J., Li R., Raes J., Arumugam M., Burgdorf K.S., Manichanh C., Nielsen T., Pons N., Levenez F., Yamada T. (2010). A human gut microbial gene catalogue established by metagenomic sequencing. Nature.

[bib69] Qin J., Li Y., Cai Z., Li S., Zhu J., Zhang F., Liang S., Zhang W., Guan Y., Shen D. (2012). A metagenome-wide association study of gut microbiota in type 2 diabetes. Nature.

[bib70] Quince C., Walker A.W., Simpson J.T., Loman N.J., Segata N. (2017). Shotgun metagenomics, from sampling to analysis. Nat. Biotechnol..

[bib71] Razgour O., Forester B., Taggart J.B., Bekaert M., Juste J., Ibáñez C., Puechmaille S.J., Novella-Fernandez R., Alberdi A., Manel S. (2019). Considering adaptive genetic variation in climate change vulnerability assessment reduces species range loss projections. Proc. Natl. Acad. Sci. U S A.

[bib72] Reese A.T., Pereira F.C., Schintlmeister A., Berry D., Wagner M., Hale L.P., Wu A., Jiang S., Durand H.K., Zhou X. (2018). Microbial nitrogen limitation in the mammalian large intestine. Nat. Microbiol..

[bib73] Rogler G., Vavricka S. (2015). Exposome in IBD: recent insights in environmental factors that influence the onset and course of IBD. Inflamm. Bowel Dis..

[bib74] Rohart F., Gautier B., Singh A., Lê Cao K.-A. (2017). mixOmics: an R package for ’omics feature selection and multiple data integration. PLoS Comput. Biol..

[bib75] Rosenberg E., Koren O., Reshef L., Efrony R., Zilber-Rosenberg I. (2007). The role of microorganisms in coral health, disease and evolution. Nat. Rev. Microbiol..

[bib76] Rosshart S.P., Vassallo B.G., Angeletti D., Hutchinson D.S., Morgan A.P., Takeda K., Hickman H.D., McCulloch J.A., Badger J.H., Ajami N.J. (2017). Wild mouse gut microbiota promotes host fitness and improves disease resistance. Cell.

[bib77] Rudman S.M., Greenblum S., Hughes R.C., Rajpurohit S., Kiratli O., Lowder D.B., Lemmon S.G., Petrov D.A., Chaston J.M., Schmidt P. (2019). Microbiome composition shapes rapid genomic adaptation of *Drosophila melanogaster*. Proc. Natl. Acad. Sci. U S A.

[bib78] Rychlik I. (2020). Composition and function of chicken gut microbiota. Animals (Basel).

[bib79] Sbahi H., Di Palma J.A. (2016). Faecal microbiota transplantation: applications and limitations in treating gastrointestinal disorders. BMJ Open Gastroenterol..

[bib80] Schmid B. (1992). Phenotypic variation in plants. Evol. Trends Plants.

[bib81] Sekula P., Goek O.N., Quaye L., Barrios C., Levey A.S., Römisch-Margl W., Menni C., Yet I., Gieger C., Inker L.A. (2016). A metabolome-wide association study of kidney function and disease in the general population. J. Am. Soc. Nephrol..

[bib82] Sheth R.U., Li M., Jiang W., Sims P.A., Leong K.W., Wang H.H. (2019). Spatial metagenomic characterization of microbial biogeography in the gut. Nat. Biotechnol..

[bib83] Singh K.M., Shah T.M., Reddy B., Deshpande S., Rank D.N., Joshi C.G. (2014). Taxonomic and gene-centric metagenomics of the fecal microbiome of low and high feed conversion ratio (FCR) broilers. J. Appl. Genet..

[bib84] Stringlis I.A., Yu K., Feussner K., de Jonge R., Van Bentum S., Van Verk M.C., Berendsen RL, Bakker P.A.H.M., Feussner I., Pieterse C.M.J. (2018). MYB72-dependent coumarin exudation shapes root microbiome assembly to promote plant health. Proc. Natl. Acad. Sci. U S A.

[bib85] Suzuki T.A., Phifer-Rixey M., Mack K.L., Sheehan M.J., Lin D., Bi K., Nachman M.W. (2019). Host genetic determinants of the gut microbiota of wild mice. Mol. Ecol..

[bib86] Vaishnava S., Yamamoto M., Severson K.M., Ruhn K.A., Yu X., Koren O., Ley R., Wakeland E.K., Hooper L.V. (2011). The antibacterial lectin RegIIIg promotes the spatial segregation of microbiota and host in the intestine. Science.

[bib87] Virtue A.T., McCright S.J., Wright J.M., Jimenez M.T., Mowel W.K., Kotzin J.J., Joannas L., Basavappa M.G., Spencer S.P., Clark M.L. (2019). The gut microbiota regulates white adipose tissue inflammation and obesity via a family of microRNAs. Sci. Transl. Med..

[bib88] Walker W.A., Floch M.H., Ringel Y., Walker W.A. (2017). Dysbiosis. The Microbiota in Gastrointestinal Pathophysiology: Implications for Human Health Prebiotics, Probiotics, and Dysbiosis.

[bib89] Wang Q., Wang K., Wu W., Giannoulatou E., Ho J.W.K., Li L. (2019). Host and microbiome multi-omics integration: applications and methodologies. Biophys. Rev..

[bib90] Wan Z.Y., Xia J.H., Lin G., Wang L., Lin V.C.L., Yue G.H. (2016). Genome-wide methylation analysis identified sexually dimorphic methylated regions in hybrid tilapia. Sci. Rep..

[bib91] Welter D., MacArthur J., Morales J., Burdett T., Hall P., Junkins H., Klemm A., Flicek P., Manolio T., Hindorff L. (2014). The NHGRI GWAS Catalog, a curated resource of SNP-trait associations. Nucleic Acids Res..

[bib92] Werren J.H., Baldo L., Clark M.E. (2008). Wolbachia: master manipulators of invertebrate biology. Nat. Rev. Microbiol..

[bib93] Wienemann T., Schmitt-Wagner D., Meuser K., Segelbacher G., Schink B., Brune A., Berthold P. (2011). The bacterial microbiota in the ceca of Capercaillie (*Tetrao urogallus*) differs between wild and captive birds. Syst. Appl. Microbiol..

[bib94] Wu H.-J., Wu E. (2012). The role of gut microbiota in immune homeostasis and autoimmunity. Gut Microbes.

[bib95] Xu Y., Zhou X. (2018). Applications of single-cell sequencing for multiomics. Methods Mol. Biol..

[bib96] Zhang G., Li C., Li Q., Li B., Larkin D.M., Lee C., Storz J.F., Antunes A., Greenwold M.J., Meredith R.W. (2014). Comparative genomics reveals insights into avian genome evolution and adaptation. Science.

[bib97] Zhao L., Zhang F., Ding X., Wu G., Lam Y.Y., Wang X., Fu H., Xue X., Lu C., Ma J. (2018). Gut bacteria selectively promoted by dietary fibers alleviate type 2 diabetes. Science.

